# Symposium on Degeneracy of the Pulp

**Published:** 1901-08

**Authors:** Eugene S. Talbot

**Affiliations:** Chicago


### SYMPOSIUM ON DEGENERACY OF THE PULP.—PRELIMINARY WORK.[1]

BY EUGENE S. TALBOT, M.D., D.D.S., CHICAGO.

MANY years ago it occurred to me that physicians could follow generally with advantage, both in pathology and physiology, the example of neurology and study of the human body from the stand-point both of its embryonic and post-uterine evolution and

---

[1] Abstract of a paper read before the Section on Stomatology, at St. Paul, Minn., June, 1901.

from the stand-point of its degeneracy as well. It was long recognized that health simply constituted a balance, and that disease meant the destruction of this balance, with resulting undue predominance of some healthy functions and the undue depression of others. Practically the same rule is followed by the chemist who controls his analytic experiments by his synthetic, and *vice versa.* Physicians and dentists generally, in studying man as an entity, view the differences between the normal and the abnormal as of kind and not of degree. To avoid this error I have studied the degenerate phases of man from the stand-point of etiology, physiology, and, lastly and most important, from the stand-point of embryology and post-uterine development periods. The result of these studies has been the general discussion of degeneracy as a phase of evolution, in "Degeneracy: Its Causes, Signs, and Results," published in London in 1898. The local degeneracies, either as an expression of general advance or its reverse, have been outlined in my works upon "The Etiology of Osseous Deformities of the Head, Face, Jaws, and Teeth," "Irregularities of the Teeth and Interstitial Gingivitis, or So-called Pyorrhœa Alveolaris," and "Degeneracy of the Alveolar Process." It is now my intention to discuss one of the most important of local degeneracies so far as dentistry is concerned,—degeneracy of the pulp. Last winter I arranged with Chicago dental surgeons that extracted teeth should be saved for me. My assistant collected from their offices every afternoon at four P.M. The teeth were then cracked open at my office, the pulps removed and placed in Müller's Fluid and one per cent. formalin before six P.M. From nineteen hundred and fifty-eight teeth obtained, one thousand and seventeen pulps were removed. Macroscopically the pulps were thus divisible: Normal; exposed, inflamed and suppurating, mummified, calcified and calculous; fungoid with exostosed roots; loose teeth abrasion; pulps destroyed with arsenic and deciduous teeth-pulps. Sound erupting third molars and bicuspids extracted in regulation had been placed in Müller's fluid ere they came under my observation.

In every case the root had to be crushed before the pulp could be detached. Pieces of the cementum almost invariably adhered to the pulp, which it required considerable force to detach. The slight adherence of pulps to the chamber was due to anatomic construction. This peculiar relation of the pulp to the walls of the canal becomes obvious when atempts are made to remove it in single-rooted teeth, after application of arsenic. The nerve-broach not infrequently carries the pulp with it in passage towards the apex. The pulp often comes out in a doubled-up contracted mass. Sometimes it comes away in pieces, and sometimes the pulp is not entirely detachable from the end of the root. The claim that pulps immediately removed from the teeth after extraction adhere to the wall of the canal is not borne out by experience. If nerve-fibres radiated from the pulp and extended from the dentine, it would be impossible to remove the pulp from its chamber. The pulp, however, cannot be removed from the end of the root without breaking; it therefore seems evident, from a macroscopic stand-point, that nerve-fibres as such do not enter the dentine. In removing the pulp it always remained *in toto* on acount of the strong connective tissue.

A point of great interest in connection with pulp adhesion to the root is not only the degeneracy of the pulp, but also of the tooth. In development of the tooth, calcification begins at the crown and extends towards the apex. Calcification takes place at the periphery of the root and extends towards the centre. A marked opening results with a large primitive organ (the pulp) until the apex is reached, when it closes. This closing often continues long after the crown has erupted. The foramen is almost completely closed. What was once a large pulp containing nerves and blood-vessels tapers down to the minutest size with but only one or two blood-vessels and nerves remaining. In the lower mammals, teeth have open ends with large pulps. These furnish sufficient nourishment to the teeth and resistance to decay. Of the one thousand and seventeen pulps collected, one hundred and thirty-one, or 11.9 per cent., were more or less mummified. While no special attempt was made to determine the extent of mummification, seventy-five per cent. of the one hundred and thirty-one seemed mummified. The remainder had varying degrees of mummification, beginning at the apical end. The extent of bloodletting at the time of extraction governed the degree of mummification. The question further arises, What amount of blood did the pulp contain at the time of extraction? Did the closure of the apical end of the root cut off the blood-supply? It is certain that teeth with mummified pulps were hard, dense, and comparatively difficult to crack. In pulps partially mummified there was a demarcation between the part filled with blood and the dried end. In the dried end mummification was complete. The dried specimen was removed from the end of the root with as much difficulty as the normal pulp, while the main part could be readily lifted out of the chamber, as there was no adhesion. The pulp can become completely mummified in from six to twelve hours. All the teeth from the same mouth were sometimes mummified. The pulps in abraded teeth had receded and been almost obliterated. Recession is sometimes followed by obliteration of the pulp-chamber. In most cases, the filling of the pulp-chamber does not follow. Blood-supply is almost if not quite exhausted. In cases where the roots were exostosed the pulps were often almost obliterated with corresponding filling in of the pulp-chamber. In other cases, mummification of the pulp occurred. There were seventeen (1.5 per cent.) calcified and calculous pulps. In some the tooth had to be completely crushed to remove the calcified mass which had adapted itself to the walls of the cavity. In others the pulp could be removed in its entirety, so complete was the calcification inside the pulp itself. There were four fungoid pulps of the one thousand and seventeen. In all four pulp-stones could be felt. The percentage of minute pulp-stones can be determined by the microscope alone.

In interstitial gingivitis, where the alveolar process has receded and the roots of the teeth have been exposed for some time, the pulp recedes and the canals become filled with dentine. Although the pulps are alive, the substance in the tubuli decomposes and the odor is more penetrating than that from dead pulps. The dentine becomes dark, and twice as much force is required to break open the tooth than in the normal variety. These teeth are brittle and fly to pieces, while the normal tooth splits lengthwise.

The pulp is a temporary organ, having attained its normal size and hence been at its best when it commences to form the dentine. The apical foramen is largest in the lower vertebrates. Pulp-stones or secondary dentine are the commencement of degeneracy. No lymphatics (Sudduth) exist in the pulp, since it is a formative as well as a degenerative organ. One hundred and thirteen, or 11.1 per cent., of the pulps examined were exposed, infected with pus, and sloughing. The inflammation sometimes extends throughout the entire pulp. Sometimes one part or side only is affected. In the case of a molar, inflammation often extends upon the side of the pulp and into one root. The other side of the pulp root or roots remains normal or is but slightly affected.

